# An Electrically Small Patch Antenna Sensor for Salt Concentration Measurement of NaCl Solution

**DOI:** 10.3390/s24196389

**Published:** 2024-10-02

**Authors:** Jinfeng Zhu, Cheng Chen, Xiao Sun, Guowen Ding, Shenyun Wang

**Affiliations:** 1 Research Center of Applied Electromagnetics, Nanjing University of Information Science and Technology, Nanjing 210044, China; 18206386323@163.com (J.Z.); cc17856077168@163.com (C.C.);; 2 School of Biological Science and Food Engineering, Chuzhou University, Chuzhou 239099, China; sunxiaonjau@126.com

**Keywords:** patch antenna sensor, salt concentration, saline solution, sensitivity

## Abstract

In this paper, a complementary split-ring resonator (CSRR)-based patch antenna is proposed as a microwave sensor to measure the salt concentration of NaCl solution. The microwave sensor consists of an RF-4 substrate, where a small copper disc is attached on the top as the radiator, a larger copper disc integrated with two CSRRs is attached on the bottom side as the finite ground plane, and a coaxial feeding port is introduced at the ground plane center. During salt concentration sensing, only the top disc is immersed into NaCl solution. The results indicate that the proposed microwave sensor can measure salt concentrations ranging from 5‰ to 35‰ with a maximum sensitivity of 0.367 (kHz/(mg/L)). The proposed microwave sensor is low-cost, low-profile, electrically small, lightweight, and easy to fabricate, and it also can be applied to other solutions’ concentration sensing.

## 1. Introduction

An intake of salt is a biological imperative, but an excessive salt intake may lead to high blood pressure and increase the risks of heart disease, stroke, and premature death [1]. Therefore, it is necessary to determine the salt contents in foods and beverages, to avoid a health risk. There are many techniques that have been proposed to determine the salt content in a food product, such as sensory evaluation and methods of chemical analysis. However, it is hard to determine an accurate salt content by sensory evaluation, and chemical methods, like titration [2], are time- and reagent-consuming, which cause environment pressures.

Alternatively, techniques based on physical principles, such as acoustic [3], nuclear magnetic resonance [4], infrared spectroscopy [5], conductivity [6], and dielectric spectroscopy [7] techniques, have been proposed to measure the solution concentration. These physical techniques feature fast and non-destructive measurement and environmental sustainability. However, they are associated with specific testing equipment, which can make the salt concentration measurement of solutions expensive and time-consuming due to the long time required for sample collection. In recent years, microwave sensing technology has garnered great attention in the field of solution concentration measurement. A variation in the complex permittivity is produced when the amount of a chemical ingredient in the solution under test (SUT) is changed. This perturbation of the dielectric permittivity affects the electromagnetic response of the microwave sensor, and it allows one to obtain information about the solution’s composition and contents.

Compared to dielectric spectroscopy, solution concentration measurement based on microwave sensors has advantages, such as rapid analysis, non-destructive testing, and environmental sustainability. Moreover, compact and low-cost measurement equipment renders it suitable for rapid solution concentration tests on spot.

Several microwave sensors have been designed based on split-ring resonators (SRRs) designed for the rapid measurement of a glucose solution’s concentration [8,9,10]. The resonant frequency of the microwave sensor was recorded for different glucose solution concentrations, and it was found that the resonant frequency increased with the increase in the glucose concentration in water. Accordingly, a graphical relationship between the measured resonance frequency and the glucose concentration was developed, which may be used to test a solution’s glucose concentration. For the measurement of the ethanol concentration, microwave sensors based on other microwave resonant structures were reported [11,12,13]. In addition, microwave sensors are widely applied in recognizing adulterated foods, such as milk [14] and edible oil [15]. A microstrip antenna sensor was proposed to detect adulteration in milk by examining the relationship between the concentration, shift in resonant frequency, and variation in reflection coefficient [14]. In Ref. [15], a two-port microstrip sensor was designed to detect the percentage of edible oil adulterated with cheaper cooking oils based on the variation in the transmission resonant frequency. These applications demonstrate the advantages of microwave sensing technology, which provides a fast, low-cost and high-sensitivity option for solution concentration measurement.

As mentioned above, the salt concentration measurement in a saline solution is perhaps the most important as it concerns the health of humans. There are many types of microwave sensors that have been proposed for the accurate measurement of the salt concentration based on resonant cavities [16,17,18], planar microwave resonators [19,20,21,22,23,24,25,26], and patch antennas [27,28,29,30,31,32,33]. Among them, the microwave sensors based on cavity resonators have a notably high sensitivity, which allows for the measurement of small changes in the salt concentration. However, the resonant cavity is hard to design and costly to manufacture, and a specific solution container or tube is needed to produce the frequency perturbation. The microwave sensors based on planar microwave resonators, such as SRRs and interdigital capacitive resonators (ICRs), also have a very high sensitivity and small size. However, despite these advantages, this microwave sensing method has to collect and encapsulate the sample within a specific solution container or channel, which makes the measurement very complicated and time-consuming. A patch-antenna-based microwave sensor has also been proposed to measure the salt concentration in a saline solution, which can be directly immersed in the saline solution for concentration measurement, instead of using specific containers, which is conducive to fast salt concentration measurement on the spot. In addition, patch antenna sensors are low-cost and easy to manufacture. However, this type of sensor usually has low sensitivity and a larger size.

In this paper, a patch-antenna-based microwave sensor with a complementary split-ring resonator (CSRR) integrated in the ground plane is proposed for salt concentration measurement. Specifically, NaCl solution is taken as the saline solution in an example, and the concentration value of interest is set from 5‰ to 35‰. The novelties of this work lie in the miniaturized structure, high sensitivity, and direct sensing without sample collection, when compared with the current microwave sensors. This paper is organized as follows. In Section 2, the structure of the patch antenna sensor is presented. In Section 3, the simulation and experimental results are illustrated. The performance evaluation of the proposed sensor is discussed in Section 4. Conclusions are drawn in Section 5.

## 2. Materials and Methods

### 2.1. Dielectric Properties of Saline Solution

The complex permittivity of NaCl solution can be expressed using the classical single Debye model fitted by Stogryn [34], Klein, and Swift [35]:

(1) $$\hat{\varepsilon }\left(\omega ,T,S\right)=\varepsilon_{0}\varepsilon_{\infty }+\frac{\varepsilon_{0}\left[\varepsilon_{s}\left(T,S\right)-\varepsilon_{\infty }\right]}{1+j\omega \tau \left(T,S\right)}-j\frac{\sigma \left(T,S\right)}{\omega }$$

where ω stands for the angular frequency of the incident electromagnetic wave, $\varepsilon_{\infty }$ is the dielectric constant at an infinite frequency, which takes the value of 4.9 in the Klein and Swift model, $\varepsilon_{s}\left(T,S\right)$, $\tau \left(T,S\right)$, and $\sigma \left(T,S\right)$ represent the static permittivity, Debye relaxation time, and conductivity of the NaCl solution, respectively, and all of them are in terms of temperature T (°C) and salt concentration S (‰), and $\varepsilon_{0}$ = 8.854 × 10^−12^ F/m is the permittivity of free space. This dielectric model has been proven to have a high accuracy in the low-frequency band. The complex permittivity given by Equation (1) can also be written as a complex form $\hat{\varepsilon }(\omega )=\varepsilon^{\prime }(\omega )-j\varepsilon^{\prime \prime }(\omega )$, and the dielectric constant and loss are given as follows:

(2) $$\varepsilon^{\prime }(\omega )=\varepsilon_{0}\left(\varepsilon_{\infty }+\frac{\varepsilon_{s}-\varepsilon_{\infty }}{1+{\left(\omega \tau \right)}^{2}}\right)$$

(3) $$\varepsilon \prime \prime (\omega )=\frac{\varepsilon_{0}\omega \tau \left(\varepsilon_{s}-\varepsilon_{\infty }\right)}{1+{\left(\omega \tau \right)}^{2}}+\frac{\sigma }{\omega }$$

By using Equations (2) and (3), it was found that the dielectric constant decreases with the increase in salinity, while the dielectric loss increases with the increase in the salt concentration [36]. However, the dielectric constant is much less sensitive to salinity than the dielectric loss.

### 2.2. Design of the Patch Antenna Sensor

As mentioned above, the dielectric constant decreases very slowly with the increase in the salt concentration of NaCl solution. Hence, the sensitivity of the microwave sensor plays a key role in acquiring accurate information on the salt concentration. In order to design a patch-antenna-based microwave sensor with high sensitivity, this demands an antenna structure operating with a high-quality factor (Q) or narrow fractional bandwidth ($B_{f}$). Antennae with narrow fractional bandwidths have found many applications in precise transponders, satellite navigation, wireless power transfer (WPT), and sensor systems. It is believed that the Q or $B_{f}$ of an antenna is limited by its size, and the relationship has been investigated by many researchers, like Chu and Wen [37,38,39,40,41,42,43],

(4) $$\max B_{f}\leq \frac{2{\left(ka\right)}^{3}}{2{\left(ka\right)}^{2}+1}\Leftrightarrow \mathrm{minQ}=\frac{1}{ka}+\frac{1}{2{\left(ka\right)}^{3}}$$

where a is the radius of the smallest sphere that completely encloses the antenna, and k=2π/λ stands for the free space wave number at the operational wavelength. Hence, to design a high-Q antenna for microwave sensing, one approach is to minimize the antenna size, which yields an electrically small antenna (ESA).

Based on the antenna theory, a CSRR-structure-based ESA is proposed in Ref. [44], and it is proven to be very electrically small, with ka < 1.0. The ESA is deformed from a monopole antenna oriented perpendicular to a finite disc ground plane and fed through a coaxial port. Next, a CSRR structure is introduced in the ground plane to generate a very low-frequency resonance. This CSRR structure consists of two split coaxial ring slots etched on the disc ground plane. The resonant frequency is mainly affected by the CSRR configuration, and it can be approximately calculated by $f_{0}=1/2\pi \sqrt{LC}$, where L and C are the inductance and capacitance of the antenna system, respectively. By introducing the CSRR structure in the finite disc ground plane, more electric and magnetic energy can be stored around the deformed monopole antenna. That is to say, a larger effective L and C are obtained, to realize a very low and narrow frequency resonance.

**Figure 1:**
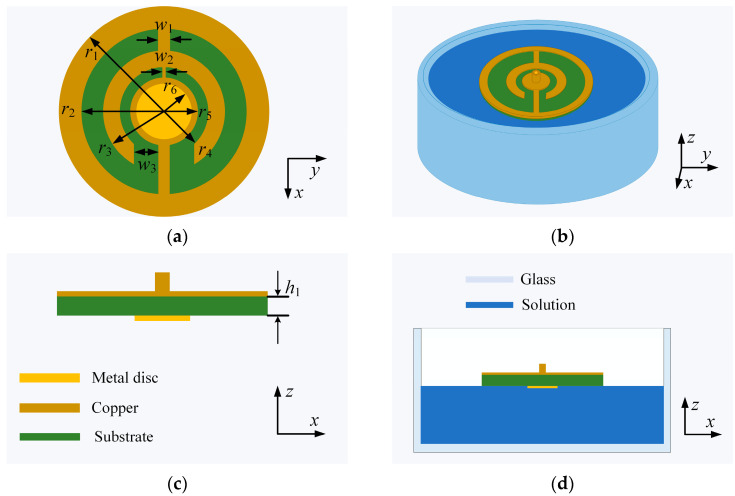
The structure of the patch antenna sensor: (**a**) top view, (**b**) 3D view in the saline solution, (**c**) side view, and (**d**) side view in the saline solution.

Inspired by the principle of the CSRR-structure-based ESA, a CSRR-structure-based patch antenna is proposed for the salt concentration measurement of NaCl solution with both high sensitivity and an electrically small size. The geometry of the proposed patch antenna structure is shown in Figure 1. It is made up of a circular dielectric substrate with a smaller metal disc and larger metal disc attached on the top and bottom, functioning as the monopole-like radiator and infinite ground plane, respectively, and a coaxial feeding port at the center of the disc ground plane. To make a low-frequency resonance and shrink the patch antenna size, two split coaxial ring slots are also introduced in the disc ground plane. Top and side views of the patch antenna structure are shown in Figure 1a,c, respectively. It should be mentioned that the patch antenna is designed under the load of a NaCl solution of 10‰ at room temperature (20 °C). The human sensory evaluation of the salt concentration ranges from 5.8 to 29.2 ‰. Higher concentrations of salt, over 29.2 ‰, may be perceived as bitter and/or sour, while very low concentrations, below 5.8 ‰, are perceived as sweet.

**Figure 2:**
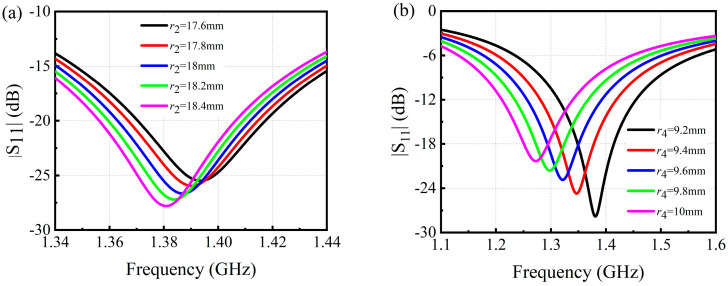
The reflectance spectra of the patch antenna sensor with (**a**) different radii of the outer CSRR, and (**b**) different radii of the inner CSRR.

In the design process, only the disc radiator is immersed in the NaCl solution for the structural optimization, as shown in Figure 1b,d. Hence, it should be pointed out that, during the salt concentration measurement, only the disc radiator of the patch antenna sensor is immersed in the saline solution as well, avoiding the CSRR-based ground plane being immersed. The substrate is FR-4 with a relative permittivity of 4.4, loss tangent of 0.02, and thickness of 1.6 mm. To obtain a patch antenna sensor with a high-Q or narrow resonant frequency band, the radius $r_{2}$ of the outer CSRR and the radius $r_{4}$ of the inner CSRR are optimized by using the CST Microwave Studio 2019, and the reflectance spectra are shown in Figure 2a,b, respectively. Finally, the patch antenna sensor is designed to operate in L band (1–2 GHz), and the final optimized geometrical parameters are listed in Table 1.

## 3. Results

### 3.1. Simulation Results

**Figure 3:**
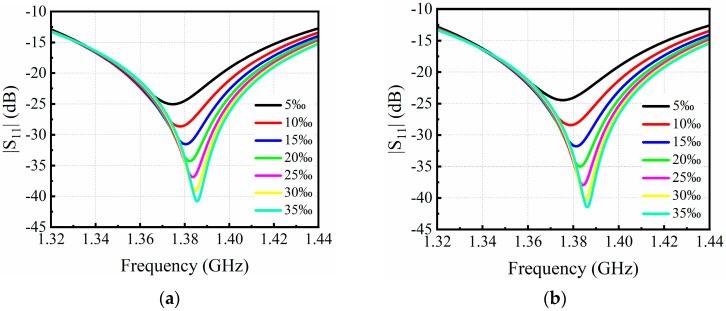

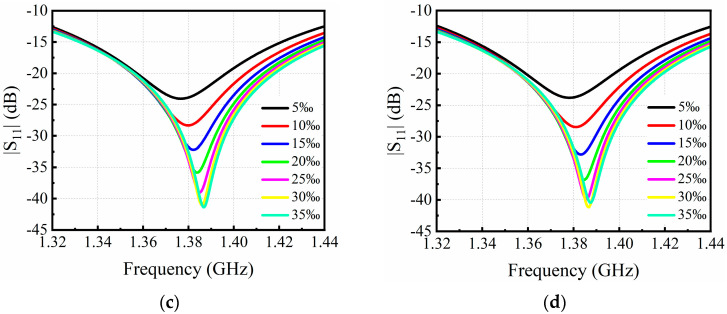
The reflectance spectra of the patch antenna sensor operating at ambient temperature of (**a**) 10 °C, (**b**) 15 °C, (**c**) 20 °C, and (**d**) 25 °C.

To validate the performance of the proposed patch-antenna-based microwave sensor for salt concentration measurement of NaCl solution, reflectance spectra were simulated with salt concentration changes at different ambient temperatures, 10 °C, 15 °C, 20 °C, and 25 °C, as shown in Figure 3a–d, respectively. The simulated salt concentration ranged from 5‰ to 35‰, resulting in a resonant frequency ranging from 1.37 to 1.38 GHz. It was found that the resonant frequency significantly increased with the increase in salt concentration at a fixed ambient temperature. With the increase in the salt concentration, the dielectric constant of the NaCl solution declined, resulting in less electric and/or magnetic energy stored. As a result, the resonant frequency moved to a higher frequency. Hence, once the resonant frequency was measured, we could obtain the salt concentration at a fixed ambient temperature.

### 3.2. Experimental Results

**Figure 4:**
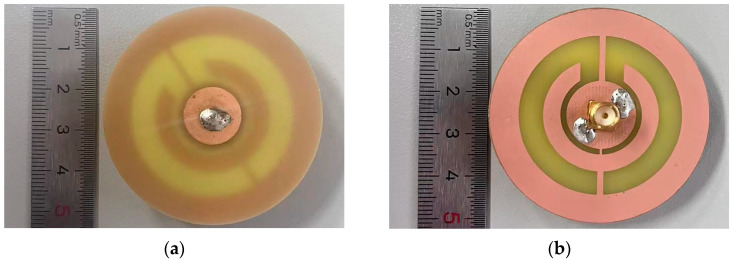
Photos of the fabricated patch antenna sensor: (**a**) top view, and (**b**) bottom view.

**Figure 5:**
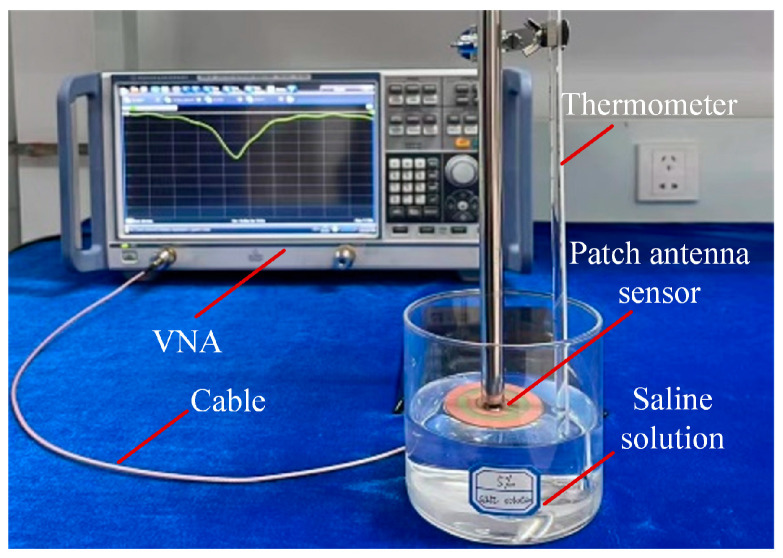
Experimental setup of the salt concentration measurement apparatus.

To validate the performance of the designed patch antenna as a microwave sensor for salt concentration sensing, a protype was fabricated and tested. Photos of the fabricated patch antenna sensor are shown in Figure 4, where a smaller copper disc is attached on the top side of the FR-4 substrate, as shown in Figure 4a, and a larger copper disc integrated with two CSRRs is fabricated on the bottom side of the FR-4 substrate. Next, the resonant frequency was measured by using a Vector Network Analyzer (VNA), R&S ZNBT40, Germany, where only the top disc radiator was immersed in the NaCl solution under testing. The experimental setup is shown in Figure 5, where NaCl solutions with salt concentrations at 5‰, 10‰, 15‰, 20‰, 25‰, 30‰, and 35‰ have been prepared. The experimental measurements were conducted at an ambient temperature of 25 °C. In order to minimize experimental errors, the measurements were repeated six times for each sample.

**Figure 6:**
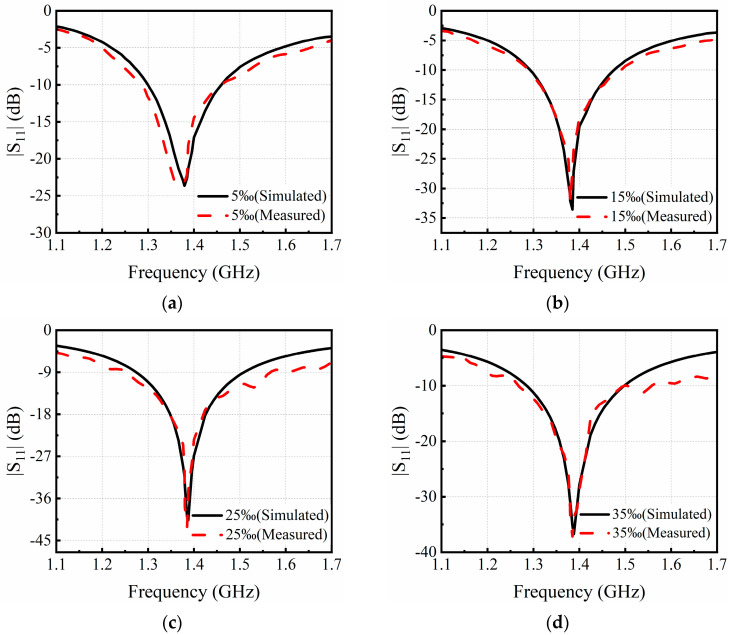
Measured and simulated reflectance spectra of the patch antenna sensor immersed in saline solutions of (**a**) 5‰, (**b**) 15‰, (**c**) 25‰, and (**d**) 35‰.

**Figure 7:**
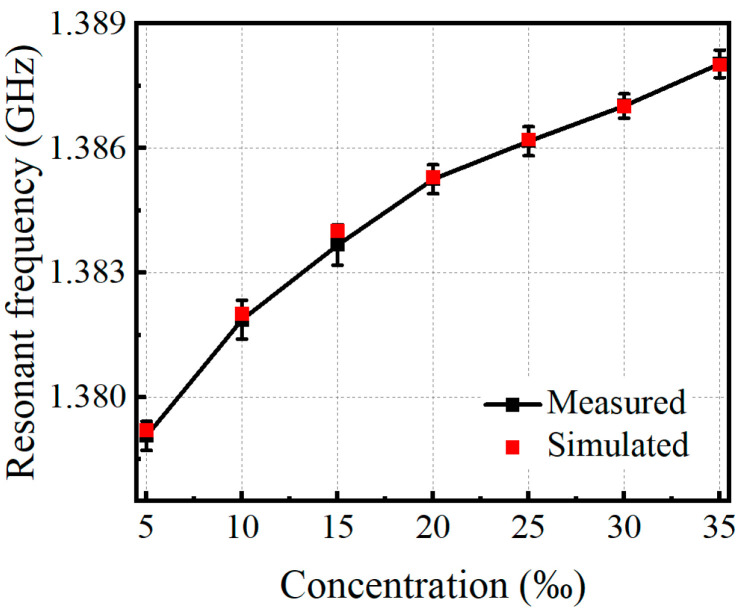
The relationship between the resonant frequency and salt concentration.

The measured and simulated reflectance spectra of the patch antenna microwave sensor for one of the measurements are plotted in Figure 6a–d with salt concentrations at 5‰, 15‰, 25‰, and 35‰, respectively. The measured resonance frequencies have good agreement with the simulated ones, indicating that the protype can acquire the accurate salt concentration of NaCl solution once it is calibrated by the simulation. When a large number of NaCl solutions are simulated or measured, the relationship between the resonant frequency and salt concentration can be obtained, as shown in Figure 7. The error bars indicate the standard deviation from the averaged amplitude values, and the absolute error of the measurement is less than 0.3%. The error is random and mainly caused by the influence of ambient temperature and operational differences in measurement. From Figure 7, it can also be found that the resonant frequency non-linearly increases with the increase in salt concentration.

## 4. Discussion

In this study, a patch antenna senor with two CSRRs integrated in the finite ground plane was designed, fabricated, and tested for the measurement of the salt concentration of NaCl solution. It has a very high sensitivity for salt concentration sensing when it is compared with the traditional patch-antenna-based microwave sensors. The sensitivity of the microwave sensor is defined as the ratio between the resonant frequency shift Δf and the increase in salt concentration ΔS.

(5) $$Sensitivity=\frac{\Delta f}{\Delta S}$$

**Figure 8:**
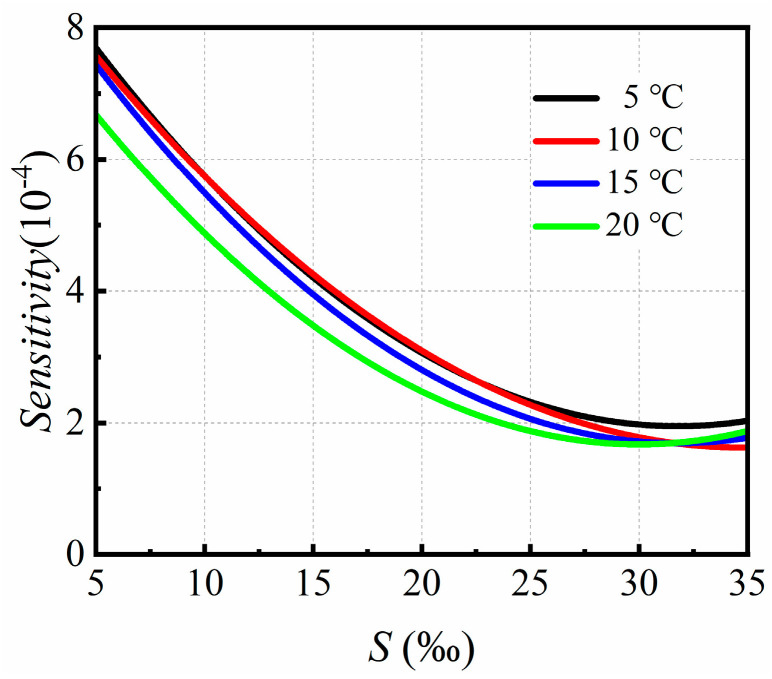
The sensitivity of the patch antenna sensor at different ambient temperatures.

It has been proven that the dielectric constant varies slowly with the increase in salt concentration [36]. Hence, the proposed patch-antenna-based microwave sensor is suitable for salt concentration sensing of NaCl solution. The sensitivity of the proposed microwave sensor at different ambient temperatures was investigated, as shown in Figure 8. It can be seen that the sensitivity tended to decrease with the increase in the salt concentration at a fixed ambient temperature. Once the salt concentration was greater than 20‰, the sensitivity stabilized or decreased very slowly. However, it should be pointed out that the sensitivity varied slightly with the variation in the ambient temperature, as shown in Figure 8.

**Figure 9:**
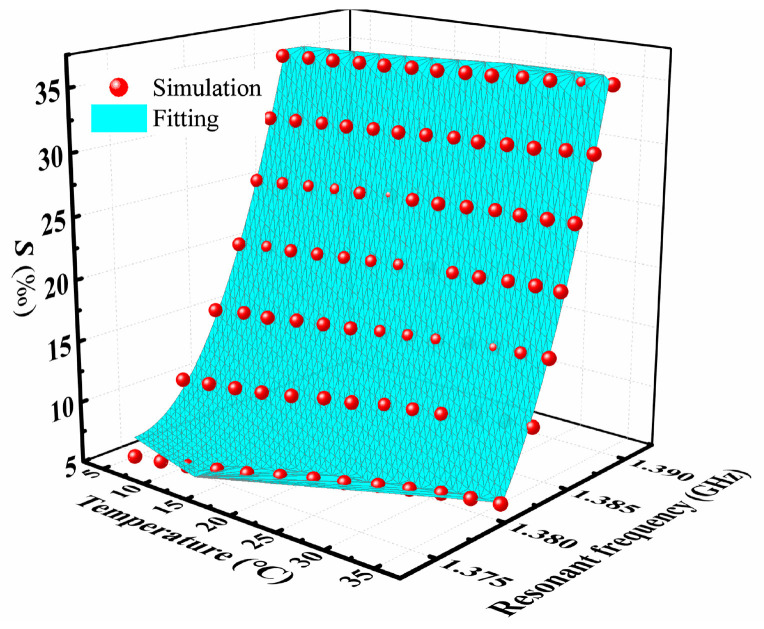
The simulated and fitted relationships among the resonant frequency, salt concentration, and ambient temperature.

Based on the simulation results, the quantitative relationship between the salt concentration (S in ‰) and resonant frequency ($f_{0}$ in GHz) at different ambient temperatures (T °C) was fitted by using the ploy-2D model with *R*^2^ = 0.99, as shown in Figure 9. The fitting formula is given by

(6) $$\begin{array}{ll} S(f_{0},T) & =(439,723.75\pm 20,005.78)+(54.74\pm 7.2)\times T-(639,959.22\pm 29,013.5)\times f_{0}-(40\pm 5)\times T\times f_{0}\\ & +(0.0023\pm 0.001)\times T^{2}+(232,847.93\pm 1.519.2)\times f_{0}^{2} \end{array}$$

When the resonant frequency is measured at a fixed temperature, one can obtain the salt concentration of the NaCl solution by using Equation (6). Moreover, the salt concentration can be directly sensed by immersing the microwave senor in the NaCl solution under testing, without sample collection and special containers or tubes. 

Finally, the proposed patch-antenna-based microwave sensor has a very electrically small size due to the introduction of the CSRR structure in the ground plane. A comparison was performed between the proposed patch-antenna-based microwave sensor and other start-of-the-art microwave sensors, as listed in Table 2. It can be seen that the proposed microwave sensor in this paper has a comparable electrically small size, but it has a greater sensitivity.

## 5. Conclusions

In this paper, a patch-antenna-based microwave sensor has been proposed to measure the salt concentration of NaCl solution. The proposed microwave sensor is low-cost, low-profile, lightweight, and easy to fabricate. Furthermore, it has a narrow bandwidth and sharp resonant frequency due to the electrically small size achieved by introducing two CSRRs in the ground plane. The proposed microwave sensor has a very high sensitivity, which is useful for measuring the salt concentration of a saline solution, as the dielectric constant varies slowly with the change in salt concentration. Finally, a prototype of the microwave sensor was fabricated and tested, and the measured results had good agreement with simulated ones. Such a microwave sensor can also be applied to the sensing of other solutions’ concentrations, such as glucose, ethanol, the polar component in fried oil, and so on.

## Figures and Tables

**Table 1:** Geometrical parameters of the patch antenna sensor.

Parameter	Value (mm)	Parameter	Value (mm)
*r*_1_	25	*w*_1_	1.4
*r*_2_	18.4	*w*_2_	0.53
*r*_3_	13.1	*w*_3_	4.2
*r*_4_	9.2	*w*_1_	1.6
*r*_5_	7.9	*w*_2_	0.1
*r*_6_	6.6	*h*_1_	1.6

**Table 2:** Comparison of the sensitivity levels and electrical sizes of the proposed microwave sensor versus start-of-the-art microwave sensors.

Ref. No.	*f*_0_ (GHz)	Sensing Parameter	Max. Sensitivity	Electrical Size
[16]	1.91	S_21_	0.015 (kHz/(mg/L))	0.7λ
[19]	2.93	S_21_	0.122 (kHz/(mg/L))	1.89λ
[20]	0.65	S_11_	0.063 (kHz/(mg/L))	0.16λ
[21]	0.25	S_11_	0.0183 (kHz/(mg/L))	0.14λ
[28]	5.65	S_11_	N/A	0.57λ
This work	1.37	S_11_	0.367 (kHz/(mg/L))	0.23λ

## Data Availability

The original contributions presented in the study are included in the article, further inquiries can be directed to the corresponding author.
